# Where Do People Vape? Insights from Twitter Data

**DOI:** 10.3390/ijerph16173056

**Published:** 2019-08-23

**Authors:** Anuja Majmundar, Jon-Patrick Allem, Tess Boley Cruz, Jennifer B. Unger

**Affiliations:** Preventive Medicine, Keck School of Medicine of the University of Southern California, Los Angeles, CA, 90032, USA

**Keywords:** vaping, locations, twitter, social media, behavior, tobacco control, electronic cigarettes, tobacco

## Abstract

*Background*: Emerging evidence suggests that exposure to secondhand and thirdhand aerosol from electronic cigarettes may have serious health risks including respiratory and cardiovascular diseases. Social media data can help identify common locations referenced in vaping-related discussions and offer clues about where individuals vape. These insights can strengthen current tobacco regulations and prioritize new policies to improve public health. This study identified commonly referenced locations in vaping-related discussions on Twitter in 2018. *Methods*: Vaping-related posts to Twitter were obtained from 1 January 2018 to 31 December 2018. Rule-based classifiers categorized each Twitter post into 11 location-related categories (social venues, living spaces, stores, modes of transportation, schools, workplaces, healthcare offices, eateries, correctional facilities, religious institutions, and miscellaneous) using a data dictionary of location-related keywords (*n* = 290,816). *Results*: The most prevalent category was social venues (17.9%), followed by living spaces (16.7%), stores (15.9%), modes of transportation (15.5%), schools (14.9%), and workplaces (11.9%). Other categories pertained to: healthcare offices (2.0%), eateries (1.2%), correctional facilities (0.7%), and religious institutions (0.4%). *Conclusion*: This study suggests that locations related to socialization venues may be priority areas for future surveillance and enforcement of smoke-free air policies. Similarly, development and enforcement of similar policies at workplaces, schools and multi-unit housing may curb exposure to secondhand and thirdhand aerosol among the public.

## 1. Introduction

Smoke-free air policies are designed to protect non-smokers from involuntary smoke exposure, denormalize smoking among youth, and encourage cessation among those who smoke [1,2,3,4,5]. Among the 42 states in the United States that have implemented public cigarette smoking bans [6], only 12 states have extended the bans to electronic cigarettes [7]. Emerging evidence suggests that potential benefits of electronic cigarette use (or vaping) includes health benefits arising from successful combustible cigarette smoking cessation [8,9]. Studies also demonstrate that vaping may pose health risks in the form of cardiovascular and respiratory diseases [10,11,12,13], and has implications for harmful secondhand [14] and thirdhand aerosol exposure for others [15,16].

Vaping regulations vary across states and are inconsistent within states. For instance, some states prohibit vaping in public locations such as restaurants and bars but may not ban vaping in private locations such as multiunit housing [17]. Other variations exist in terms of type of products regulated (e.g., electronic cigarettes, hookahs), substances (cannabis, e-liquids), type of locations (public vs. private), and level of jurisdiction (statewide vs. county vs. institutional policies) [17,18,19,20]. These inconsistencies may have implications for enforcement of vaping bans. For instance, stealth vaping, defined as vaping discreetly in locations where e-cigarettes are prohibited, is becoming more common to circumvent regulations [21]. Stealth vaping is prevalent among youth, who vape in school [22], and among adults, who vape at their workplaces, bars/nightclubs, airports, and at movie theatres [21], Social media data, in this context, provides an opportunity to conduct large-scale surveillance of organic, real-time conversations about locations referenced in vaping-related discussions that offer clues about where individuals vape, to offer actionable insights for strengthening current regulations and prioritizing new policies in the interest of public health.

Twitter is used by 24% of US adults (24% of women, 23% of men, 24% of white individuals, 26% of African American individuals, and 20% of Hispanic individuals), with 46% of users on the platform daily [23]. About 32% of adolescents also report using Twitter daily [24]. In the current study, we demonstrate the utility of collecting data from Twitter to document and describe the types of locations referenced in vaping-related posts.

## 2. Materials and Methods

Data Collection

Twitter (https://twitter.com/) posts containing 26 vaping-related terms (e.g., ‘vape’, ‘e-cigarettes’, ‘juul,’ etc.) drawn from previous research [25,26,27], were obtained from 1 January 2018 to 31 December 2018 using Twitter’s Streaming Application Program Interface (API). The initial sample consisted of 11,148,880 Twitter posts. We used rigorously tested Python scripts to exclude retweets, non-English Twitter posts, Twitter posts from bot accounts [28] Twitter posts from accounts posting a higher than normal frequency of posts, duplicate posts that were not retweets and promotional posts were also excluded. Twitter posts were normalized through lemmatization, converted to lower case, and features such as punctuation, special characters, hyperlinks and hashtags were removed [22]. A subset of Twitter posts containing 11 categories of locations derived from location-related keywords was filtered from this initial sample using Python scripts. In other words, rule-based classification script using Python 3.7 searched for presence of at least one keyword per Twitter post. During this process we tested our Python scripts to ensure that the automated filtering was accurate. The location-related keywords (e.g., school, bedroom, home, house, etc.) were generated from past work identifying locations referenced in the text of tweets [29] from open source libraries [30], and from other online resources [31,32]. The resulting list of keywords was manually curated to also include contextually relevant location keywords such as ‘classroom’ and ‘party’. The analytic sample consisted of 290,816 Twitter posts from 204,724 users. Please refer to Figure S1 for a flowchart of these procedures.

The analyses relied on public, anonymized data, adhered to the terms and conditions, terms of use, and privacy policies of Twitter. This study was performed under Institutional Review Board approval from the authors’ university. No Twitter posts were reported verbatim in this report to protect the privacy of the users. Python code and associated search keywords are publicly available on a repository (see https://github.com/anujamajmundar/VapingLocations).

## 3. Results

A total of 11 location-based categories were identified in the sample. Table 1 shows the most prevalent themes and associated illustrative keywords. The most prevalent category was socialization venues (17.9%), followed by living spaces (16.7%), stores (15.9%), modes of transportation (15.5%), schools (14.9%), and workplaces (11.9%). Other locations pertained to: healthcare offices (2.0%), eateries (1.2%), correctional facilities (0.7%), and religious institutions (0.4%).

## 4. Discussion

This study identified common locations referenced in Twitter discussions related to vaping in 2018. Social venue was the most commonly referenced category. This raises concerns about normalization of vaping in public spaces. Regulatory agencies could consider raising public awareness about the health risks of exposure to secondhand aerosol from electronic cigarettes. Vaping in groups with peers, in general, may also risk normalizing the behavior and undermine associated long- and short-term health risks. Future education campaigns addressing pro-vaping social norms can incorporate location-based contexts to demonstrate effects on vulnerable populations sharing a given space.

Vaping in private locations such as living spaces, workplaces, and educational institutions creates substantial health risk for family members, peers and neighbors in the form of second and third hand aerosol exposure [14,15,16]. Education campaigns tailored for families, employees or students are needed to increase awareness about the dangers of second and thirdhand aerosol exposures. A recent study found that e-cigarette health risk education efforts through advertising, media coverage and interpersonal discussion can enhance perceived harmfulness of secondhand aerosol exposure [33]. Evidence also suggests that employees often report an inadequate understanding of their workplace vaping policies [34]. Educational programs at workplaces, as such, can enhance adherence to vaping regulations among employees. Given the findings from this study, these programs may need to be prioritized to curb vaping at work.

Similar to prior research, this study found that vaping was taking place on school grounds during school hours [22,35]. Many schools in the United States are in the process of responding to the epidemic of vaping among their students through awareness initiatives and school-level bans of vape products [36]. Findings from this study suggest that vaping on school premises is still a common occurrence and in need of regulation.

References to vaping while in cars and other modes of *transportation* were common in the data and raises concern over road safety and secondhand and thirdhand aerosol exposure in confined spaces. Educational campaigns need to raise these concerns to motorists to help protect passengers. Future communication strategies could utilize the amber alert systems to curb vaping in public and private modes of transportation similar to messages that raise the issue of driving under the influence. Future research may investigate ways in which vaping in restricted-use locations may influence the perception of already existing anti-smoking regulations.

## 5. Limitations

This study drew data from Twitter and findings may not generalize to other social media platforms. Findings may not represent data from individuals with private Twitter accounts. The time range of the data is January to December 2018, and findings may not generalize to other years. While over multiple locations were utilized to understand vaping-related locations, specific references to names of locations (e.g., name of a restaurant or club) may not be captured in this study.

## 6. Conclusions

This study identified common locations referenced in Twitter discussions related to vaping in 2018. Locations associated with *social venues* may be priority areas for future surveillance and enforcement of no-smoking policies. Similarly, development and enforcement of similar policies at workplaces, schools and multi-unit housing may curb exposure to secondhand and thirdhand aerosol among the public. As tobacco regulation continues to evolve with the growing use of electronic cigarettes, social media data can help inform policymakers about gaps in tobacco regulations by highlighting predominant locations referenced with vaping.

## Figures and Tables

**Table 1:** Illustrative keywords and prevalence of each theme in the sample.

No.	Locations	Illustrative Keywords	%
1.	Social venues	Game, Party, Park, Beach	17.9
2.	Living space	Home, Bedroom, Hotel	16.7
3.	Stores	Shop, Store, Market, Gym	15.9
4.	Modes of transportation	Car, Drive, Train, Bus	15.5
5.	School	School, Class, College	14.9
6.	Workplace	Work, Job, Workplace	11.9
7.	Healthcare offices	Doctor, Dentist, Drugstore	2.0
8.	Eateries	Restaurant, Café, Boba	1.2
9.	Correctional facility	Court, Prison, Jail	0.7
10.	Religious institutions	Church, Chapel, Temple	0.4
11.	Miscellaneous	Place, Town, Downtown, Neighborhood	13.5

## Supplementary Materials

The following are available online at https://www.mdpi.com/1660-4601/16/17/3056/s1, Figure S1: Data management procedures.
