# Supplementary figures and images for: Where Do People Vape? Insights from Twitter Data
Source: Int J Environ Res Public Health. 2019 Aug 23;16(17):3056. doi: 10.3390/ijerph16173056 (PMC6747114; doi:10.3390/ijerph16173056)

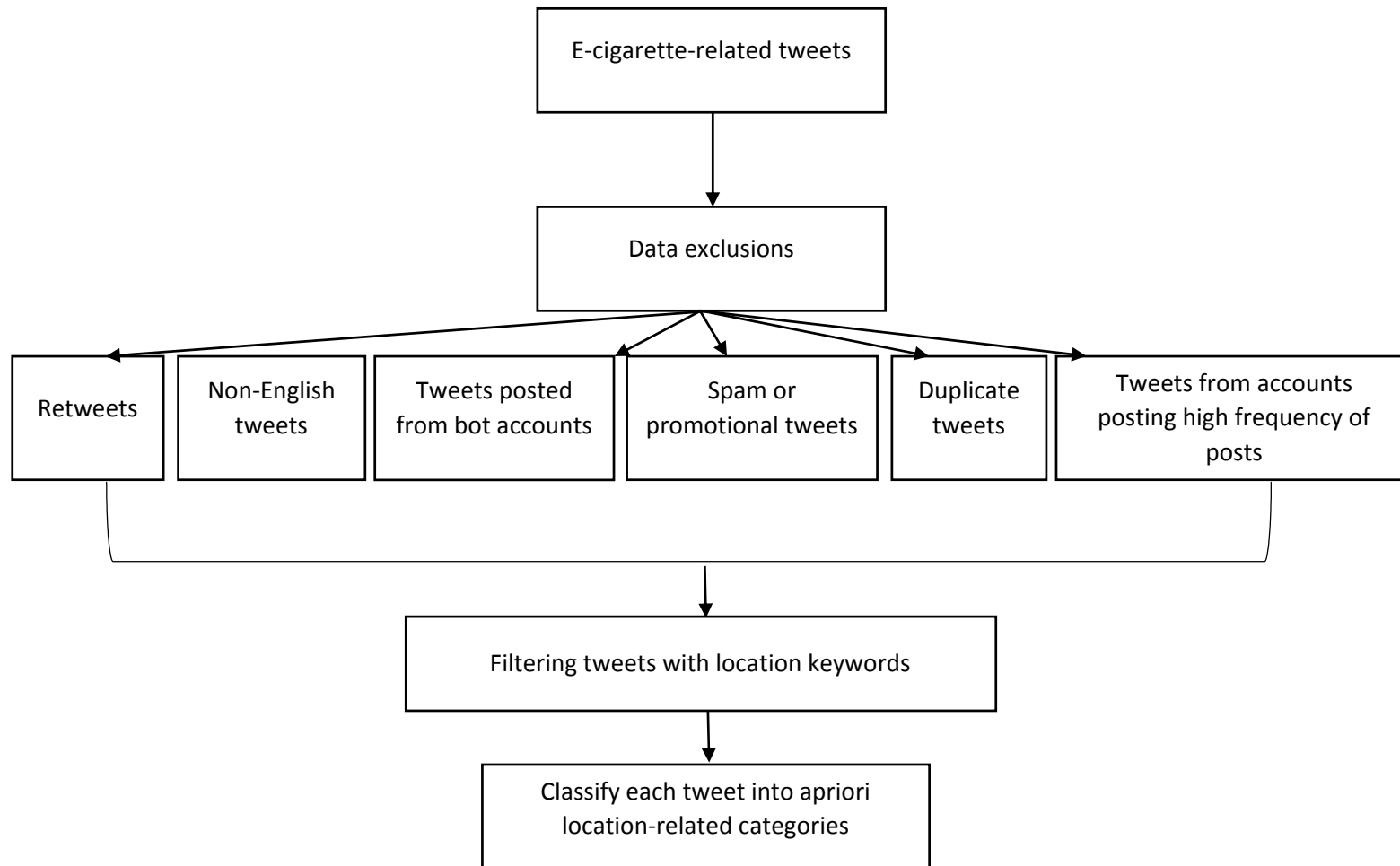

**Figure S1.** Data management procedures.

Supplement: Supplementary file 1 [file ijerph-16-03056-s001.pdf]
